# Viscosity-Dependent Shrinkage Behavior of Flowable Resin Composites

**DOI:** 10.3390/polym17243292

**Published:** 2025-12-11

**Authors:** Nadja Jeconias, Peter Fischer, Tobias T. Tauböck

**Affiliations:** 1 Department of Conservative and Preventive Dentistry, Center for Dental Medicine, University of Zurich, Plattenstrasse 11, 8032 Zurich, Switzerland; tobias.tauboeck@zzm.uzh.ch; 2 Department of Health Sciences and Technology, Institute of Food, Nutrition and Health, ETH Zurich, 8092 Zurich, Switzerland; peter.fischer@hest.ethz.ch

**Keywords:** flowable resin composites, viscosity, polymerization shrinkage, shrinkage stress

## Abstract

Flowable resin composites are extensively used in restorative dentistry, where linear polymerization shrinkage and the resulting shrinkage stress are critical for clinical success. This study investigated the relationship between viscosity, linear polymerization shrinkage, and shrinkage stress in flowable resin composite materials. Two low-flow resin composites (Beautifil Flow Plus F00, Estelite Universal Flow SuperLow), two medium-flow resin composites (Tetric EvoFlow, Estelite Universal Flow Medium), and two high-flow resin composites (Beautifil Flow F10, Estelite Universal Flow High) were examined. Viscosity (*n* = 3) of the unset materials was determined using a cone–plate rheometer. The composites were photoactivated for 20 s at 1226 mW/cm^2^, and linear polymerization shrinkage (*n* = 8) and shrinkage stress (*n* = 8) of 1.5 mm-thick specimens were recorded in real time for 5 min using a custom-made linometer and stress analyzer, respectively. Data were analyzed with Kruskal–Wallis rank tests followed by Conover post hoc tests, and Spearman correlation analyses were conducted to assess relationships between parameters (α = 0.05). A significant negative correlation was observed between viscosity and shrinkage stress (r = −0.943, *p* = 0.017). Beautifil Flow F10 exhibited the significantly lowest viscosity (14.60 ± 0.17 Pa·s) and the highest shrinkage stress (0.83 ± 0.14 MPa) among the materials, whereas low-flow composite Estelite Universal Flow SuperLow showed the lowest shrinkage stress (0.65 ± 0.10 MPa). Linear shrinkage ranged from 1.89 ± 0.13% to 3.18 ± 0.21%, but was not correlated with viscosity or stress (*p* > 0.05). In conclusion, viscosity critically influences polymerization-induced shrinkage stress development in flowable resin composites. Higher-viscosity flowable composites might be beneficial regarding stress build-up during polymerization compared with high-flow composites.

## 1. Introduction

Flowable resin composites have become essential materials in restorative dentistry. They exhibit enhanced flow characteristics compared to conventional composite materials, allowing for better adaptation to cavity walls, ease of placement, and improved handling properties [1]. Initially, flowable resin composites were developed as cavity bases and liners [2]. However, these materials expanded substantially over the past decades and are now categorized by manufacturers into high-flow, medium-flow, and low-flow classifications [3].

The introduction of varying viscosities has broadened the range of clinical applications for flowable composites, enhancing their versatility in treatment. The selection of an appropriate material viscosity is influenced by the clinician’s preference and specific clinical requirements [4]. This variability offers significant advantages, enabling their application not only in small cavities but also in larger restorations due to enhanced mechanical properties of more viscous formulations [3,5]. As a result of their increased viscosity and superior mechanical properties, low-flow resin composites are indicated for posterior restorations and load-bearing areas [6,7,8], while high-flow resin composites are recommended for small cavities and preventive procedures such as fissure sealings, where ease of flow and application are essential [8,9,10]. Manufacturers modify polymer formulations by varying filler content and resin matrix composition [11,12]. This approach aims to adapt flowability while simultaneously reducing polymerization shrinkage and optimizing the mechanical performance of the material [13,14].

One of the primary factors influencing the performance of flowable resin composites is viscosity, which is predominantly determined by the filler content [15,16]. The interaction between viscosity and filler content not only governs the material’s flow behavior, but also affects polymerization shrinkage [4,13]. Due to their reduced filler content, flowable resin composites have been shown to exhibit higher polymerization shrinkage and shrinkage stress compared with conventional resin composites [17,18], which may result in potential complications such as microleakage and marginal gaps [19]. This lower filler loading is further associated with inferior mechanical performance, including reduced compressive strength, and decreased wear resistance [20]. The matrix composition influences resin composite rheology through the type and proportion of monomers [21]. High-molecular-weight monomers generally increase viscosity, but do not directly determine filler loading [22,23], while low-molecular-weight diluent monomers reduce matrix viscosity [24,25,26]. Monomer composition also affects polymerization kinetics and filler–matrix coupling, and together with filler characteristics dictates the composite’s overall rheological behavior [4,27]. However, information regarding the impact of viscosity on shrinkage behavior of flowable resin composites remains, as yet, limited [28,29]. Despite the growing variety of flowable resin composites available on the market, the clinical implications of their viscosity differences remain insufficiently understood. Although manufacturers continuously introduce new formulations with modified rheological profiles, clinicians still lack clear evidence on how viscosity affects polymerization-induced shrinkage and shrinkage stress—two parameters that are critical for marginal integrity and restoration longevity. Furthermore, the interrelationship between viscosity, filler content, and monomer composition is often complex and cannot be inferred solely from manufacturer specifications. A more detailed investigation of how viscosity relates to shrinkage stress behavior is needed to provide clinicians with a sound basis for selecting suitable materials.

Therefore, the aim of this study was to investigate the relationship between viscosity, linear polymerization shrinkage, and shrinkage stress across a range of commercially available flowable resin composite materials. The null hypothesis stated that there are no differences between the flowable resin composites in terms of viscosity, linear polymerization shrinkage, and shrinkage stress.

## 2. Materials and Methods

### 2.1. Composite Materials

Six flowable resin composite materials categorized by manufacturers were used in this study: two low-flow resin composites [Beautifil Flow Plus F00 (Shofu, Kyoto, Japan) and Estelite Universal Flow SuperLow (Tokuyama, Tokyo, Japan)], two medium-flow resin composites [Tetric EvoFlow (Ivoclar Vivadent, Schaan, Liechtenstein) and Estelite Universal Flow Medium (Tokuyama, Tokyo, Japan)], and two high-flow resin composites [Beautifil Flow F10 (Shofu, Kyoto, Japan) and Estelite Universal Flow High (Tokuyama, Tokyo, Japan)]. Detailed material information is provided in Table 1.

### 2.2. Viscosity Measurements

The viscosity of the flowable resin composites was measured using a rheometer (MCR 501, Anton Paar, Graz, Austria) in a cone–plate geometry, using micro-roughened 25 mm plates (PP25, Anton Paar, Graz, Austria) to avoid slip, and a gap of 0.05 mm. The cone angle was 1 degree. The flow chamber was fixed to the stage and connected to a temperature control unit. The temperature of the chamber was set to 22 °C and maintained at this level. The resin composite material was poured on the measuring geometry, and the upper plate was lowered to the gap size. The whole chamber was covered with an opaque, light-proof apparatus preventing any exposure to external light. A flow curve test was then performed in a shear rate regime from 0.1 to 1000 s^−1^. The test was repeated three times per composite material, with a new specimen prepared each time.

### 2.3. Linear Polymerization Shrinkage

**Figure 1:**
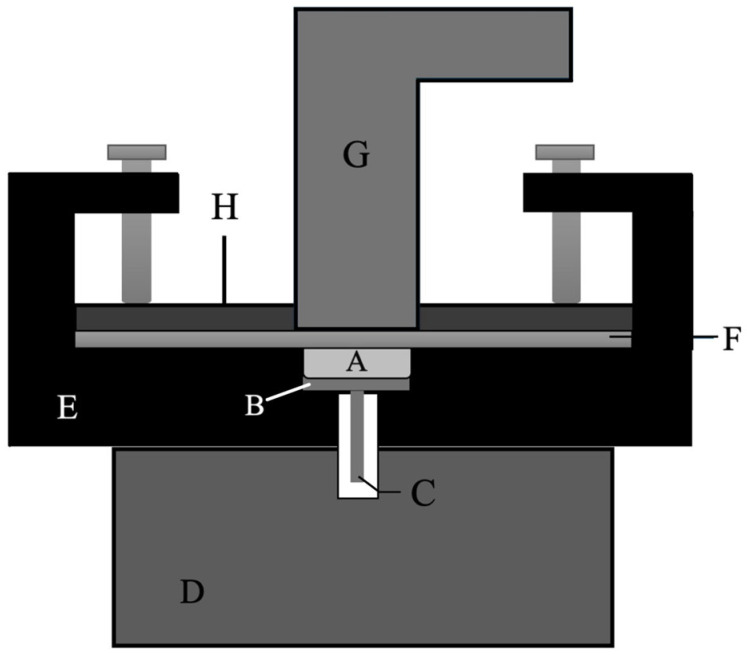
Schematic illustration of the setup (linometer) used for linear shrinkage. A—resin composite specimen, B—aluminum platelet, C—diaphragm, D—infrared measuring sensor, E—metal frame, F—glass plate, G—light curing tip, H—aluminum plate.

Linear shrinkage was measured using a custom-made linometer (Figure 1), as previously described in the literature [30]. The linometer was constructed of a solid metal frame consisting of aluminum. An amount of 42 mm^3^ of composite material was applied on a thin platelet, which contained a vertical diaphragm on its bottom. The platelet was loosely placed upon the solid metal frame of the linometer, and the diaphragm reached into the zone of the infrared measuring sensor. The specimen was covered with a sandblasted (50 μm Al_2_O_3_; Renfert, Hilzingen, Germany) and silanized (Monobond Plus; Ivoclar Vivadent, Schaan, Liechtenstein) 1 mm-thick glass plate and leveled to a thickness of 1.5 mm. All specimens were photoactivated for 20 s using an LED light-curing unit (Bluephase PowerCure, Ivoclar Vivadent, Schaan, Liechtenstein) with a 10 mm light emission window. The light guide tip was positioned directly above the glass plate, and the output irradiance was 1226 mW/cm^2^, as measured and verified periodically during the experiment with a calibrated FieldMax II-TO power meter (Coherent, Santa Clara, CA, USA). Experimental testing was conducted inside a temperature-controlled chamber set to 25 °C, simulating intraoral conditions after rubber dam placement [31]. The real-time measurements (*n* = 8 per material) were performed at a sampling frequency of 5 Hz during 5 min from the start of photoactivation.

### 2.4. Polymerization Shrinkage Stress

**Figure 2:**
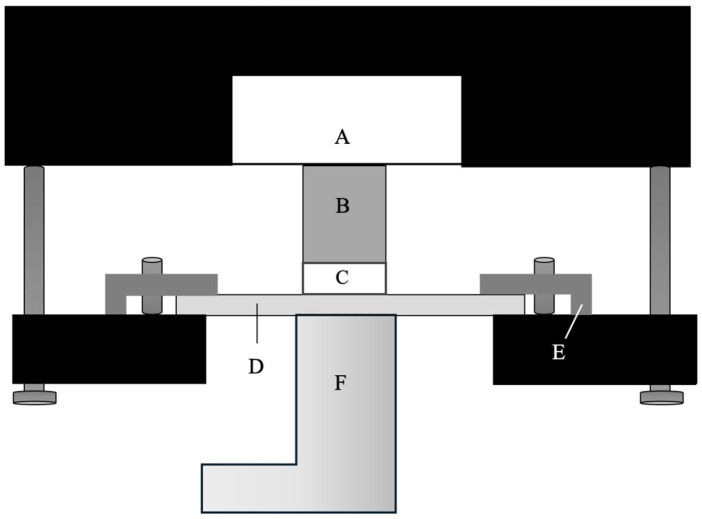
Schematic illustration of the setup used for shrinkage force. A—load cell, B—metal cylinder, C—resin composite specimen, D—glass plate, E—holder of glass plate, F—light curing tip.

A customized stress analyzer was used to measure polymerization shrinkage stress (Figure 2), based on principles as also described in detail previously [32,33]. The device consisted of a semi-rigid load cell (PM 11-K, Mettler, Greifensee, Switzerland; instrument compliance: 0.4 μm/N), to which a metal cylinder with a diameter of 6 mm was attached. The composite material (42 mm^3^) was applied to the upper side of the cylinder and compressed to a thickness of 1.5 mm by a glass plate, which was fixed to the lower side of the device. The test assembly with composite disks of 6 mm diameter and 1.5 mm thickness exhibited a configuration factor of 2.0. Both the surface of the glass plate and the metal cylinder were sandblasted with 50-μm aluminum oxide (Renfert, Hilzingen, Germany) to improve adhesion, cleaned with air water spray for 30 s and after air-drying, coated with a universal primer (Monobond Plus, Ivoclar Vivadent, Schaan, Liechtenstein). The composite materials were photoactivated for 20 s through the glass plate as described above. The forces resulting during polymerization shrinkage were detected by the load cell at a sampling frequency of 5 Hz. Real-time measurements (*n* = 8 per material) were performed for 5 min from the start of photoactivation. Nominal stress (in MPa) was calculated by dividing the measured force by the bonded surface area.

### 2.5. Statistical Analysis

Due to substantial heteroscedasticity, the non-parametric Kruskal–Wallis rank test, followed by Conover tests was used to compare the different groups. The *p*-values were adjusted for multiple testing according to Holm’s procedure. Spearman correlation coefficients and corresponding *p*-values were calculated to assess the strength and significance of the association between viscosity, linear shrinkage, and shrinkage stress. All statistical analyses were performed using the statistical software R 4.4.0 [34], including the package tidyverse [35]. The overall level of significance was set to α = 0.05.

## 3. Results

**Figure 3:**
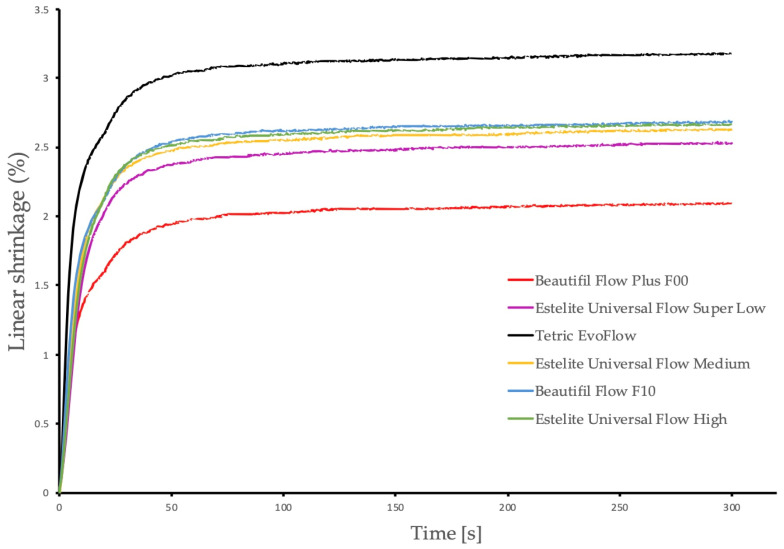
Time-dependent mean linear shrinkage curves of all tested flowable resin composites.

**Figure 4:**
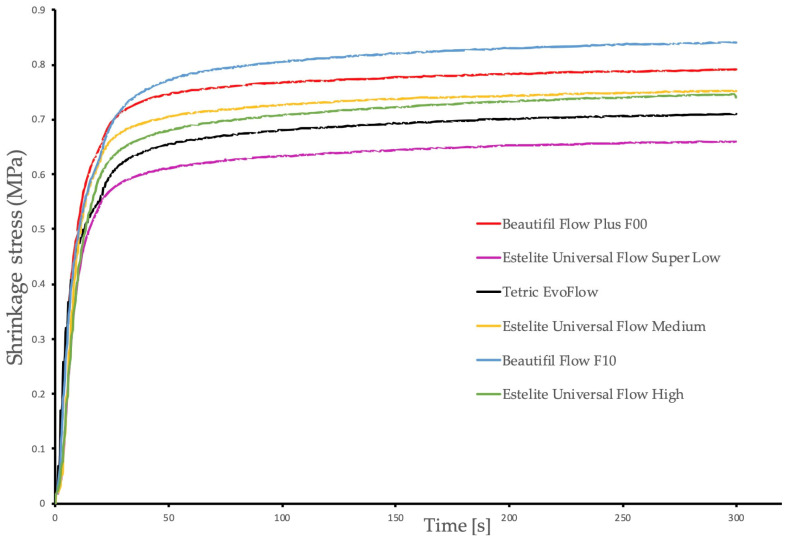
Time-dependent mean shrinkage stress curves of all tested flowable resin composite.

Figure 3 and Figure 4 demonstrate the evolution of linear polymerization shrinkage and polymerization shrinkage stress as a function of time, respectively. Table 2 displays the linear shrinkage and shrinkage stress measured at the end of the 5 min observation period, as well as the viscosity of the investigated flowable resin composites at a shear rate of 10 s^−1^.

The significantly lowest linear shrinkage was generated by the low-flow composite Beautifil Flow Plus F00, whereas the significantly highest linear shrinkage was generated by the medium-flow composite Tetric EvoFlow. Low-flow composite Estelite Universal Flow SuperLow, medium-flow composite Estelite Universal Flow Medium, and the two high-flow composites Estelite Universal Flow High and Beautifil Flow F10 ranked in between, with no significant differences in linear shrinkage among them.

The lowest shrinkage stress was generated by the low-flow composite Estelite Universal Flow SuperLow, which did not differ significantly from Tetric EvoFlow, Estelite Universal Flow Medium, Beautifil Flow Plus F00 and Estelite Universal Flow High. The high-flow composite Beautifil Flow F10 generated significantly higher shrinkage stress compared with Estelite Universal Flow SuperLow.

The significantly highest viscosity at a shear rate of 10 s^−1^ was measured for Tetric EvoFlow followed by the low-flow composite Estelite Universal Flow SuperLow, the medium-flow composite Estelite Universal Flow Medium, the low-flow composite Beautifil Flow Plus F00 and the high-flow composite Estelite Universal Flow High. The significantly lowest viscosity was revealed for high-flow Beautifil Flow F10.

A significant negative correlation was found between median shrinkage stress and median viscosity at a shear rate of 10 s^−1^ (r = −0.943, *p* = 0.017). No significant correlations were revealed between linear shrinkage and viscosity (r = 0.058, *p* = 0.913), and between linear shrinkage and shrinkage stress (r = 0.174, *p* = 0.742).

## 4. Discussion

The present study investigated the effect of viscosity on linear polymerization shrinkage and polymerization shrinkage stress of six different flowable resin composite materials. Significant differences were identified among the flowable resin composites in terms of viscosity, linear polymerization shrinkage, and polymerization shrinkage stress, which led to the rejection of the null hypothesis.

A significant inverse correlation was observed between shrinkage stress and median viscosity, indicating that low-viscosity flowable materials are more prone to develop higher polymerization shrinkage stress. The flowable resin composite with the lowest viscosity measured, Beautifil Flow F10, exhibited the highest shrinkage stress, whereas Estelite Universal Flow SuperLow, a highly viscous composite, exhibited the lowest shrinkage stress. One explanation might be differences in the materials’ filler contents, which affect viscosity [4,36,37]. Estelite Universal Flow SuperLow has one of the highest filler loadings (70 wt%) among the tested materials, which might have contributed to comparatively reduced shrinkage stress. In contrast, Beautifil Flow F10 with the lowest filler loading (53 wt%), exhibited the lowest measured viscosity along with the highest polymerization shrinkage stress. Similar findings have been reported by Tauböck et al. demonstrating that composites with higher filler loading and greater viscosity exhibited significantly lower shrinkage stress compared to low-viscosity materials [30]. An increased filler content reduces the overall proportion of the resin matrix, which in turn decreases the number of reactive methacrylate groups, limiting volumetric shrinkage and internal stress development [38].

The observed viscosity-stress relationship can be further understood through the material’s capacity to undergo viscous flow prior to the gel point. Low-viscosity composites allow greater molecular mobility during the pre-gel phase, enabling stress relaxation but also permitting higher volumetric shrinkage [25]. In contrast, highly viscous materials reach the gel point earlier, which limits their ability to flow during polymerization and therefore results in a reduced potential for stress relaxation [4]. This interpretation aligns with established rheological principles governing the interplay between flow behavior, gelation kinetics, and stress development in resin-based composites [39].

In addition to these rheological considerations, measurements conditions influence the results. Viscosity was assessed at a shear rate of 10 s^−1^, a value commonly employed in dental materials research because it approximates the shear conditions encountered during clinical placement and manipulation of flowable composites [4]. However, as viscosity is inherently shear-rate dependent and most resin composites exhibit shear-thinning behavior [36], different shear rates may alter absolute viscosity values and affect the strength of the correlations observed [40]. Future studies examining viscosity across multiple shear rates would therefore help verify the robustness of these findings and more accurately reflect the dynamic conditions of clinical handling.

Besides viscosity and rheological flow, filler content and the elastic behavior of the measurement system can also influence shrinkage stress development. Although higher filler content generally reduces polymerization shrinkage, it simultaneously increases the elastic modulus of the composite [13,41], which may enhance stress formation according to Hooke’s law. While rigid testing devices presuppose ideal elastic behavior according to Hooke’s Law, semi-rigid systems, as the linometer used in the present study, allow limited axial deformation during curing and thereby better simulate the partial compliance of dentin substrates [42]. Previous studies have reported a positive correlation between filler content and shrinkage stress [43,44,45]. However, these investigations utilized near-zero compliance (highly rigid) test setups incorporating negative feedback mechanisms to fully compensate for axial specimen deformation during measurements. In such rigid systems, the elastic modulus appears to be the predominant factor governing shrinkage stress formation [44]. In contrast, when semi-rigid or more compliant measuring devices are employed, shrinkage stress development is predominantly governed by the extent of polymerization shrinkage rather by the elastic modulus [45]. Variations in instrument compliance may, therefore, account for conflicting observations reported in the literature regarding the influence of filler content on shrinkage stress [44]. Our results show a general decrease in polymerization shrinkage stress with increasing filler content, likely reflecting the higher compliance of the testing system and the dynamic interrelation of material properties influencing stress development. Semi-rigid stress analyzers offer a more realistic simulation of intraoral conditions and are useful for characterizing the shrinkage behavior of resin-based composites under clinically realistic constraints [45,46].

In addition to filler content and filler size [4,47], monomer composition also plays a substantial role in determining viscosity. Low-flow resin composites frequently contain bisphenol-A-glycidyldimethacrylate (Bis-GMA), a rigid, high-molecular-weight monomer, which increases viscosity due to extensive hydrogen bonding and restricted chain mobility [26,48]. To decrease viscosity, manufacturers incorporate low-viscosity diluent monomers, such as triethylene glycol dimethacrylate (TEGDMA). While such diluents effectively reduce viscosity, they may increase polymerization shrinkage because of their high proportion of reactive methacrylate groups [49]. An exception to the general trend was observed in Estelite Universal Flow Medium. Although this material contains a higher filler content than Estelite Universal Flow SuperLow, it exhibited a lower viscosity and a higher polymerization shrinkage stress. This indicates that viscosity is not solely governed by filler loading, but can be substantially influenced by the monomer composition, the ratio of diluent to base monomers, and material-specific filler–matrix interactions. In this case, the specific monomer formulation likely reduced viscosity despite higher filler content, while a greater proportion of reactive methacrylate groups may have contributed to the increased shrinkage stress [4]. Beside monomer composition, the degree of double-bond conversion (DC) represents another material-related parameter that affects shrinkage behavior. A lower DC, as well as an increased filler content, can also contribute to a reduction in polymerization-induced shrinkage stress and should therefore be considered when interpreting the results [32,50].

According to the manufacturer-reported technical documentation, Tetric EvoFlow is stated not to contain TEGDMA [51]. Low-viscosity monomers, such as TEGDMA, are commonly incorporated in flowable materials to reduce viscosity due to their low molecular weight and flexible molecular structure [40]. The measured viscosity of Tetric EvoFlow deviated from the manufacturer’s specifications, which may be attributed to the material’s complex and proprietary monomer-filler formulation. In fact, the organic matrix of Tetric EvoFlow appears more viscous than that of other flowable composites included in this study, which is consistent with the general principle that monomer rigidity, hydrogen-bonding capacity, and molecular mobility collectively determine the final viscosity of a resin blend [4]. Because the exact monomer ratios are not disclosed by the manufacturer, no definitive conclusion regarding the predominance of specific monomers can be drawn; however, the observed behavior aligns with the expected trends for systems containing higher proportions of rigid aromatic monomers. It should be acknowledged that the conclusions regarding monomer composition and its influence on viscosity are based on manufacturer-reported formulations, as no chromatographic or spectroscopic verification was performed in the present study. Therefore, these interpretations remain partly speculative. The final viscosity of a composite resin is thus a balance between monomer rigidity, hydrogen bonding, and the proportion of reactive diluents [49]. As demonstrated by Lovell et al., replacing 25–50% of Bis-GMA with TEGDMA can reduce viscosity by up to 80%, but also leads to higher polymerization shrinkage and reduced control over polymer network formation [25].

Finally, differences in viscosity and shrinkage stress development may also be influenced by the characteristics of the filler system, including the specific surface area, degree of silanization, and the overall filler–matrix interaction. Therefore, differences in viscosity observed among the tested materials may not solely result from variations in monomer composition. These factors should be considered alongside monomer composition when interpreting viscosity-related findings [4,52,53].

A potential limitation of the present study is that not all flowable composites may necessarily follow the same trends observed in our results. Certain materials incorporate chain-transfer agents (CTAs), which are known to markedly reduce polymerization-induced shrinkage stress without affecting viscosity. The evaluation of such CTA-containing composites would therefore be an interesting avenue for future research.

## 5. Conclusions

In conclusion, viscosity plays a decisive role in the development of polymerization-induced shrinkage stress in flowable resin composites. Compared with high-flow materials, more viscous flowable composites may offer advantages in reducing stress development during polymerization. Clinically, understanding how viscosity influences polymerization-induced shrinkage stress may help clinicians select flowable composites that reduce interfacial stress and improve marginal adaptation. Future research should investigate CTA-containing materials and examine polymerization kinetics and degree of conversion to clarify the mechanisms identified in this study. Additionally, future studies are needed to determine whether increasing the viscosity of flowable resin composites is more effectively achieved through adjustments in filler content or through modifications in resin composition. Systematic comparisons of these strategies would help identify which approach offers the most predictable improvements in rheological behavior and shrinkage stress reduction. Moreover, studies assessing how higher shrinkage stress affects marginal integrity under clinically relevant conditions would provide further insight into the translational relevance of these findings.

## Figures and Tables

**Table 1:** Manufacturer-reported composition and technical specifications of the investigated resin composite materials used in the study.

Material	Composition	Filler Size (μm)	Filler Content (wt%/vol%)	Lot No./Shade	Manufacturer
Beautifil Flow Plus F00	Matrix: Bis-GMA ^1^, TEGDMA ^2^, CQ ^3^ Filler: AFS-glass ^4^, SiO_2_ ^5^, Al_2_O_3_ ^6^, colorant	1–3	67/47	062331/A2	Shofu, Kyoto, Japan
Estelite Universal Flow SuperLow	Matrix: POE ^7^, Bis-MPEPP ^8^, EDBO ^9^, UVP ^10^ Filler: spherical SiO_2_, TiO_2_ ^11^ ZrO_2_ ^12^ nanoparticles	mean: 0.2	70/56	073E33/A2	Tokuyama, Tokyo, Japan
Tetric EvoFlow	Matrix: Bis-GMA, UDMA ^13^, D3DMA ^14^, TPO ^15^, CQ, EDAB ^16^ Filler: YbF_3_ ^17^, barium glass, ZrO_2_-SiO_2_ mixed oxide, prepolymerized filler particles	0.1–15.5	68/46	Z06F2Z/A2	Ivoclar Vivadent, Schaan, Liechtenstein
Estelite Universal Flow Medium	Matrix: POE, Bis-MPEPP, EDBO, UVP Filler: spherical SiO_2_, TiO_2_ ZrO_2_ nanoparticles	mean: 0.2	71/57	101E73/A2	Tokuyama, Tokyo, Japan
Beautifil Flow F10	Matrix: Bis-GMA, TEGDMA, CQ Filler: AFS-glass, Al_2_O_3_, SiO_2_, colorants	mean: 0.8	53/33	062392/A2	Shofu, Kyoto, Japan
Estelite Universal Flow High	Matrix: POE, Bis-MPEPP, EDBO, UVP Filler: spherical SiO_2_, TiO_2_ ZrO_2_ nanoparticles	mean: 0.2	69/55	079EZ3/A2	Tokuyama, Tokyo, Japan

^1^ Bis-GMA: bisphenol-A-glycidyldimethacrylate; ^2^ TEGDMA: triethylene glycol dimethacrylate; ^3^ CQ: camphorquinone; ^4^ AFS-glass: alumino-fluoro-borosilicate glass; ^5^ SiO_2_: silicon dioxide; ^6^ Al_2_O_3_: aluminumoxid; ^7^ POE: poly(oxyethylene); ^8^ Bis-MPEPP: bis [2-(methacryloyl-oxy) ethoxy] phenyl propane; ^9^ EDBO: 1,2-ethanediol bis(oxy-2,1-ethanediyl) dimethacrylate; ^10^ UVP: 2-(2H-benzotriazol-2-yl)-4-methylphenol; ^11^ TiO_2_: titanium dioxide; ^12^ ZrO_2_: zirconium oxide; ^13^ UDMA: urethane dimethacrylate; ^14^ D3DMA: 1,10-decanediol dimethacrylate; ^15^ TPO: trimethylbenzoyl diphenylphosphine oxide; ^16^ EDAB: ethyl 4-(dimethylamino)benzoate; ^17^ YbF_3_: ytterbium trifluoride.

**Table 2:** Mean values (±standard deviation) of linear polymerization shrinkage and polymerization shrinkage stress at the end of the 5 min observation period, and viscosity measured at a shear rate of 10 s^−1^ for all tested flowable resin composites. Different uppercase letters indicate statistically significant differences between materials (*p* < 0.05).

Manufacturer Classification	Material	Linear Shrinkage (%)	Shrinkage Stress (MPa)	Viscosity at Shear Rate of 10 s^−1^ (Pa·s)
Low-flow	Beautifil Flow Plus F00	1.89 (0.13) C	0.75 (0.12) AB	37.87 (1.70) D
Low-flow	Estelite Universal Flow SuperLow	2.53 (0.20) B	0.65 (0.10) B	60.23 (1.60) B
Medium-flow	Tetric EvoFlow	3.18 (0.21) A	0.70 (0.08) AB	67.17 (2.55) A
Medium-flow	Estelite Universal Flow Medium	2.63 (0.14) B	0.73 (0.09) AB	48.00 (0.70) C
High-flow	Beautifil Flow F10	2.68 (0.21) B	0.83 (0.14) A	14.60 (0.17) F
High-flow	Estelite Universal Flow High	2.67 (0.19) B	0.80 (0.09) AB	28.10 (0.27) E

## Data Availability

The original contributions presented in this study are included in the article. Further inquiries can be directed to the corresponding author.
